# Genome-Wide Association Analysis of Ischemic Stroke in Young Adults

**DOI:** 10.1534/g3.111.001164

**Published:** 2011-11-01

**Authors:** Yu-Ching Cheng, Jeffrey R. O’Connell, John W. Cole, O. Colin Stine, Nicole Dueker, Patrick F. McArdle, Mary J. Sparks, Jess Shen, Cathy C. Laurie, Sarah Nelson, Kimberly F. Doheny, Hua Ling, Elizabeth W. Pugh, Thomas G. Brott, Robert D. Brown, James F. Meschia, Michael Nalls, Stephen S. Rich, Bradford Worrall, Christopher D. Anderson, Alessandro Biffi, Lynelle Cortellini, Karen L. Furie, Natalia S. Rost, Jonathan Rosand, Teri A. Manolio, Steven J. Kittner, Braxton D. Mitchell

**Affiliations:** * Department of Medicine; ‡ Department of Neurology, and; § Department of Epidemiology and Public Health, University of Maryland School of Medicine, Baltimore, Maryland 21201; † Department of Neurology, Veterans Affairs Medical Center, Baltimore, Maryland 21201; ** Department of Biostatistics, University of Washington, Seattle, Washington 98195; †† Center for Inherited Disease Research, Johns Hopkins University School of Medicine, Baltimore, Maryland 21224; ‡‡ Department of Neurology, Mayo Clinic, Jacksonville, Florida 32224; §§ Department of Neurology, Mayo Clinic, Rochester, Minnesota 55905; *** Laboratory of Neurogenetics, National Institute of Aging and; §§§§ National Human Genome Research Institute, National Institutes of Health, Bethesda, Maryland 20892; ††† Center for Public Health Genomics; ‡‡‡ Department of Public Health Sciences, and; §§§ Department of Neurology, University of Virginia, Charlottesville, Virginia 22908; **** Center for Human Genetic Research and; †††† Department of Neurology, Massachusetts General Hospital, Boston, Massachusetts 02114; ‡‡‡‡ Program in Medical and Population Genetics, Broad Institute, Cambridge, Massachusetts 02142

**Keywords:** epidemiology, genetics, brain infarction, FMNL2

## Abstract

Ischemic stroke (IS) is among the leading causes of death in Western countries. There is a significant genetic component to IS susceptibility, especially among young adults. To date, research to identify genetic loci predisposing to stroke has met only with limited success. We performed a genome-wide association (GWA) analysis of early-onset IS to identify potential stroke susceptibility loci. The GWA analysis was conducted by genotyping 1 million SNPs in a biracial population of 889 IS cases and 927 controls, ages 15–49 years. Genotypes were imputed using the HapMap3 reference panel to provide 1.4 million SNPs for analysis. Logistic regression models adjusting for age, recruitment stages, and population structure were used to determine the association of IS with individual SNPs. Although no single SNP reached genome-wide significance (*P* < 5 × 10^−8^), we identified two SNPs in chromosome 2q23.3, rs2304556 (in *FMNL2; P* = 1.2 × 10^−7^) and rs1986743 (in *ARL6IP6; P* = 2.7 × 10^−7^), strongly associated with early-onset stroke. These data suggest that a novel locus on human chromosome 2q23.3 may be associated with IS susceptibility among young adults.

Age-related stroke is a common debilitating disease that ranks among the leading causes of death in the United States (Rosamond *et al.* 2008). Ischemic stroke (IS) comprises approximately 85% of all strokes. While hypertension, cigarette smoking, and diabetes are well-established risk factors for IS, family and twin studies provide evidence that IS aggregates within families (Flossmann *et al.* 2004), thus implicating a genetic contribution to stroke susceptibility.

Efforts to identify the genetic determinants of IS have had limited success. One strategy to enhance the chances of identifying stroke susceptibility genes is to study stroke cases that are more likely to have a strong genetic predisposition. Such cases might include those with a strong family history of stroke or those experiencing a stroke at a relatively young age. Such strategies have been successful in identifying genes associated with early-onset forms of other complex diseases, including breast cancer (Walsh *et al.* 2006), Parkinson’s disease (Lesage *et al.* 2008), obesity (Hinney *et al.* 2007; Scherag *et al.* 2010), inflammatory bowel disease (Imielinski *et al.* 2009), and myocardial infarction (Kathiresan *et al.* 2009). Evidence from twin (Brass *et al.* 1992; Brass *et al.* 1998; Hassan and Markus 2000) and familial aggregation studies (Schulz *et al.* 2004), in fact, supports a stronger genetic contribution to early-onset than later-onset stroke. We have previously reported in our own study of young-onset stroke that the proportion of cases reporting a family history of stroke increases with decreasing age of the proband (MacClellan *et al.* 2006).

We have performed a case-control study of IS in subjects ages 15–49 years. In this report, we present results of a genome-wide association (GWA) analysis based on 1.4 million SNPs genotyped and/or imputed across the genome for their association with risk of IS.

## Materials and Methods

### Study population

The Genetics of Early Onset Stroke (GEOS) Study is a population-based, case-control study designed to identify genes associated with early-onset ischemic stroke and to characterize interactions of identified stroke genes and/or SNPs with environmental risk factors. Participants were recruited from the greater Baltimore-Washington area in four different periods: Stroke Prevention in Young Women-1 (SPYW-1) conducted from 1992 to 1996, Stroke Prevention in Young Women-2 (SPYW-2) conducted from 2001 to 2003, Stroke Prevention in Young Men (SPYM) conducted from 2003 to 2007, and Stroke Prevention in Young Adults (SPYA) conducted in 2008. From these samples, we identified a total of 921 cases and 941 controls that consented to having their DNA used for genetic studies of stroke. This study was conducted with the consent of all study participants and was approved by the University of Maryland at Baltimore Institutional Review Board.

### Definitions of cases and controls

“Case participants” were hospitalized with a first cerebral infarction identified by discharge surveillance from one of the 59 hospitals in the greater Baltimore-Washington area and direct referral from regional neurologists. IS with the following characteristics were excluded from participation: stroke occurring as an immediate consequence of trauma; stroke within 48 hr after a hospital procedure, stroke within 60 days after the onset of a nontraumatic subarachnoid hemorrhage, and cerebral venous thrombosis. Additional exclusions for these genetic analyses are listed in Table 1. All cases had neuroimaging that was consistent with cerebral infarction, although neuroimaging was not used for case ascertainment. The abstracted hospital records of cases were reviewed and adjudicated for IS subtype by a pair of neurologists according to previously published procedures (Johnson *et al.* 1995; Kittner *et al.* 1998), with disagreements resolved by a third neurologist. The IS subtype classification system retains information on all probable and possible causes, and it is reducible to the more widely used TOAST system (Adams *et al.* 1993) that assigns each case to a single category. All cases had age of first stroke between 15 and 49 years and were recruited within three years of stroke.

**Table 1:** Subject exclusion criteria for genetic analysis in GEOS study

Exclusion Criteria
1. Known single-gene or mitochondrial disorders recognized by a distinctive phenotype; *e.g.*, cerebral autosomal dominant arteriopathy with subcortical infarcts and leukoencephalopathy (CADASIL), mitochondrial encephalopathy with lactic acidosis and stroke-like episodes (MELAS), homocystinuria, Fabry disease, or sickle cell anemia
2. Mechanical aortic or mitral valve at the time of index stroke
3. Untreated or actively treated bacterial endocarditis at the time of the index stroke
4. Neurosyphilis or other CNS infections
5. Neurosarcoidosis
6. Severe sepsis with hypotension at the time of the index stroke
7. Cerebral vasculitis by angiogram and clinical criteria
8. Post-radiation arteriopathy
9. Left atrial myxoma
10. Major congenital heart disease
11. Cocaine use in the 48 hr prior to the index stroke

“Control participants” without a history of stroke were identified by random-digit dialing. Controls were balanced to cases by age and region of residence in each study and were additionally balanced for race in SPYW-2 and SPYM. Traditional stroke risk factors and other study variables, including age, race/ethnicity, history of hypertension, diabetes, myocardial infarction (MI), and current smoking status (defined as use within one month prior to event for cases and at a comparable reference time for controls), were also collected during a standardized interview.

### Genotyping

Genomic DNA was isolated from a variety of sample types, including cell line (55.2%), whole blood (43.1%), mouth wash (0.4%), and buccal swab (0.05%). Whole-genome amplification (Qiagen REPLI-g kit, Valencia, CA) was used to obtain sufficient DNA for genotyping in 1.3% of samples. The distribution of sample types did not differ significantly between cases and controls (56.3% cell lines in cases *vs.* 54.1% in controls; 41.5% whole blood in cases *vs.* 44.9% in controls). Samples were genotyped at the Johns Hopkins Center for Inherited Disease Research (CIDR), and genotyping was performed using the Illumina HumanOmni1-Quad_v1-0_B BeadChip (Illumina, San Diego, CA). Case and control samples were balanced across the plates, and self-identified whites and African Americans were placed on different plates. Samples of 50 self-reported whites were also placed on African American plates for quality control. All study samples, including 39 blind duplicates (19 whites and 20 African Americans), were plated and genotyped together with 42 HapMap control samples, including 26 Utah residents with ancestry from northern and Western Europe (CEU) and 16 Yoruba (YRI) samples, and all samples were processed together in the lab. Allele cluster definitions for each SNP were determined using Illumina BeadStudio Genotyping Module version 3.3.7, Gentrain version 1.0, and the combined intensity data from all released samples. Genotypes were not called if the quality threshold (Gencall score) was below 0.15.

Genotypes of a total of 1827 study individuals (99% of attempted samples) were released by CIDR, and all had a genotype call rate greater than 98%. Genotyping concordance rate was 99.996% based on study duplicates. We excluded 11 individuals from analysis due to unexpected duplicates, gender discrepancy, or unexpected relatedness, leaving a total of 1816 individuals (889 cases and 927 controls) in the final analysis. A total of 1,014,719 SNPs were released by CIDR (99.83% of attempted). Genotypes were not released for SNPs that had call rates less than 85%, a cluster separation value of less than 0.2, more than 1 HapMap replicate error, more than a 5% (autosomal) or 6% (X) difference in call rate between sexes, more than 0.3% male AB frequency (X), or more than a 11.3% (autosomal) or 10% (XY) difference in AB frequency. Individual SNPs were excluded postanalysis if they had excessive deviation from Hardy-Weinberg equilibrium (HWE) proportions (*P* < 1.0 × 10^−7^) or genotype call rates less than 95%. Departure from HWE was assessed by χ^2^ test among controls only and among each ethnic group separately. For this report, only SNPs having minor allele frequencies (MAF) greater than 1% and SNPs passing HWE filtering in both genetically defined (see *Statistical Analysis* below) European ancestry (EA) and African ancestry (AA) populations were included (N = 784,766 SNPs).

### Imputation

Imputation was performed for genetically defined EA cohort (N = 946) and AA cohort (N = 733) separately using BEAGLE v3.3 software (Browning and Browning 2009). Genetically defined EA participants were those within two standard deviations (SD) of the mean of the first and second principal components for subjects self-identified as “white,” and similarly, genetically defined AA participants were those within two SD of the mean of the first and second principal components for those self-identified as “African American.” The combined samples of CEU and Tuscans from Italy (TSI) from the HapMap Phase 3 populations were used as the reference panel for EA cohort imputation. A combination of five HapMap Phase 3 populations, CEU, TSI, YRI, Luhya in Webuye, Kenya (LWK), and African ancestry in southwest United States (ASW), was used as reference panel for AA cohort imputation, in accordance with the recommendation that a mixture of populations be used as the reference panel for African Americans (Hao *et al.* 2009). All SNPs failing the composite quality filter were removed [*i.e.* SNPs with missing call rate ≥ 5%, SNPs with Mendelian errors in three HapMap trios > 0, HWE *P* < 1 × 10^−4^, and SNPs with more than one discordant genotype call across the 89 duplicated study samples (39 blind duplicates and 50 white duplicates plated on African American plates]. All remaining SNPs having a MAF ≥ 1% in each respective cohort were used as input SNPs for imputation. A total of 1,387,466 SNPs were obtained via imputation, which included 532,595 and 582,186 input SNPs for the EA and AA cohorts, respectively. Imputed SNPs that have an estimated squared correlation between the estimated allele dosage and the true allele dosage (dosage r^2^) < 0.3 were removed postanalysis. The dosage r^2^ can be derived from arguments found in Appendix 1 of Browning and Browning (2009). For this study, we supplemented 784,766 SNPs genotyped by the Illumina Omni1-Quad chip with 637,233 imputed SNPs with r^2^ > 0.3 for a total of 1,421,999 SNPs in the final genome-wide association report.

To assess the performance of imputation, analysis was performed of a masked set of SNPs on chromosome 1. We first masked a randomly selected set of 20% of all Omni1 array SNPs on chromosome 1 that passed the preimputation filters. The masked SNPs were then removed from input files for imputation, and then imputation was performed using the same procedures as described previously. The imputed genotypes at the “masked” SNPs were then compared with the experimental (typed) genotypes from Omni1 GWAS data to assess the accuracy of imputation. The SNP concordance rates, which were defined as fraction of identical genotypes between the most likely imputed and observed, were 0.9858 for EA and 0.9805 for AA (filtering imputed genotypes at posterior probability ≥ 0.9). The correlations between observed *vs.* expected allele frequencies for all imputed SNPs were also very high (0.99967 for EA and 0.99937 for AA). The plot of the observed allelic frequency *vs.* expected allelic frequency from imputation is shown in supporting information, Figure S1.

### Statistical analysis

Summary statistics of study characteristics were calculated and expressed as unadjusted means ± SDs (SD) or percentages, stratified by case status. We compared cases and controls for difference in means using *t*-tests (for continuous variables) or difference in proportions by χ^2^ tests (for categorical variables) to obtain the unadjusted two-sided *P* values. Logistic regression with case/control status as the outcome and baseline characteristics as predictors was used to obtain the age- and sex-adjusted *P* values. All summary statistics of study characteristics were obtained using the STATA statistical package (version 9.2; StataCorp, College Station, TX).

GWA analysis was performed using a logistic regression model to test for associations of each SNP with IS under an additive model (for imputed SNPs, estimated allelic dosage was used). A t-score was used to assess the significance of the beta coefficient (β) for the SNP, and the odds ratio (OR) was derived by exponentiation of the beta coefficient. All estimates were obtained after adjusting for the effect of age, recruitment periods (three indicator variables), and population structure. Population structure was estimated using multidimensional scaling (MDS) analysis on the matrix of genome-wide identical-by-state (IBS) pairwise distances, which were estimated based on 127,901 typed (not imputed) SNPs that were selected for having MAF > 5%, SNP call rates > 95%, and not being in linkage disequilibrium with other selected SNPs (*i.e.* pair-wise LD between any two SNPs was constrained to be r^2^ < 0.2). The first 10 dimensions of population structure were extracted using MDS, but only the first dimension was significantly associated with case status and included as a covariate in the association analysis. Similar to imputation, study subjects were divided into genetically defined EA and AA groups for association analyses based on population structure analysis. EA participants were defined as those failing within two SD of the mean of the first and second components for self-reported “white” subjects, and AA participants were defined as falling within two SD of the mean of the first and second components for self-reported “African American” subjects. Association analyses were conducted in the total population, adjusting for the first MDS component, as well as for genetically defined EA and AA groups separately. Genome-wide significance level was set at α = 5.0 × 10^−8^ after Bonferroni correction for multiple testing. All analyses were performed using existing programs implemented in PLINK version 1.07 (Purcell *et al.* 2007). Power calculations (using Quanto v1.2.4) indicated that our sample size of 889 cases and 927 controls provided 80% power to detect ORs ranging 1.55–2.30 for allele frequencies ranging 10–50% at an α = 5.0 × 10^−8^.

We also reviewed previous genome-wide association studies on IS. We identified one gene, *NINJ2*, reported previously to be genome-wide significantly associated with stroke risk in a large-scale meta-analysis (CHARGE consortium) (Ikram *et al.* 2009). To replicate the reported associations with *NINJ2*, a logistic regression model was conducted under an additive genetic assumption to obtain beta coefficients (and odds ratios) and 95% confidence intervals after adjusting the same set of covariates described above. We first tested whether the two SNPs previously reported by CHARGE (rs12425791 and rs11833579) were associated with young-onset stroke in our sample. Because of our *a priori* hypothesis that each SNP would be associated with young-onset IS, we did not adjust for multiple comparisons in this analysis (α = 0.05). We also conducted an agnostic screening of all genotyped/imputed SNPs located within *NINJ2* to identify the most-significant SNP from the gene based on empirical *P* values to control for the number of SNPs tested. Empirical *P* values for the most-significant SNPs were obtained by permuting the case/control status 1000 times to obtain the null distribution of the maximum T statistics (over all SNPs within the gene) and calculating the number of times the maximum test statistics exceeded the observed test statistic. Analyses were conducted using a set-based permutation procedure implemented in PLINK 1.07 (Purcell *et al.* 2007).

## Results

### Characteristics of study population

A total of 1816 GEOS study participants, including 889 cases and 927 controls, were included in the analysis. Characteristics of study participants are summarized in Table 2. The mean age was 41.3 years for cases and 39.6 years for controls (*P* < 0.001). The population is primarily composed of two self-reported race groups, white (54.5%) and African American (40.4%), with the remaining 5.1% of individuals comprising other races, including Chinese, Japanese, other Asians, and other unspecified. There were more males than females among both cases and controls. Cases were more likely than controls to report having prevalent hypertension, diabetes, and myocardial infarction and to being current smokers.

**Table 2:** Population characteristics by case-control status

Characteristic	Case (n = 889)	Control (n= 927)	*P**^a^*
Age (mean ± SD, years)	41.3 ± 6.9	39.6 ± 6.8	<0.001
Female (%)	41.5	43.6	0.37
Self-reported ethnicity (%)			
White	52.42	56.42	0.22
African American	42.41	38.51	
Other	5.17	5.07	
Subtype (%)			
Cardioembolic	20.0	—	—
Large artery	7.1		
Lacunar	16.1		
Other known cause	6.5		
Undetermined cause	50.3		
Hypertension (%)	42.7	19.2	<0.001
Diabetes mellitus (%)	16.7	5.1	<0.001
Angina/myocardial infarction (%)	5.3	0.7	<0.001
Current smoker (%)	42.5	28.6	<0.001

a Unadjusted *P* values for age, sex, and race; age and sex-adjusted *P* values for other characteristics.

### Genome-wide association of early-onset IS

**Figure 1:**
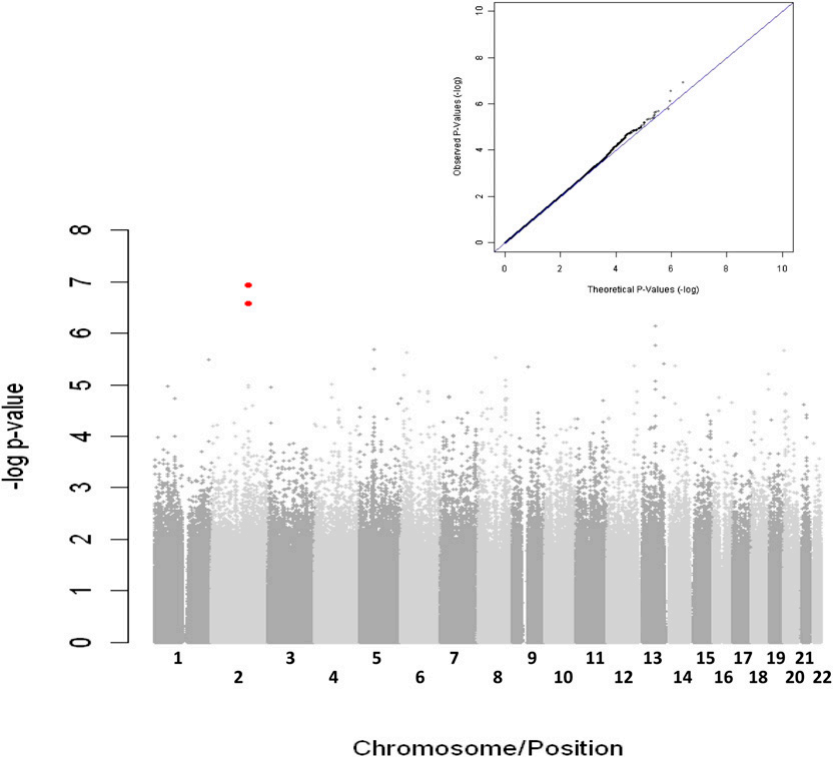
Negative log10 of genome-wide *P* values from logistic regression model and a quantile-quantile (Q-Q) plot (upper right) of the distribution of these observed test statistics against expected distribution in overall samples. Red dots represent the two most significant SNPs in chromosome 2q (rs2304556 and rs1986743).

**Figure 2:**
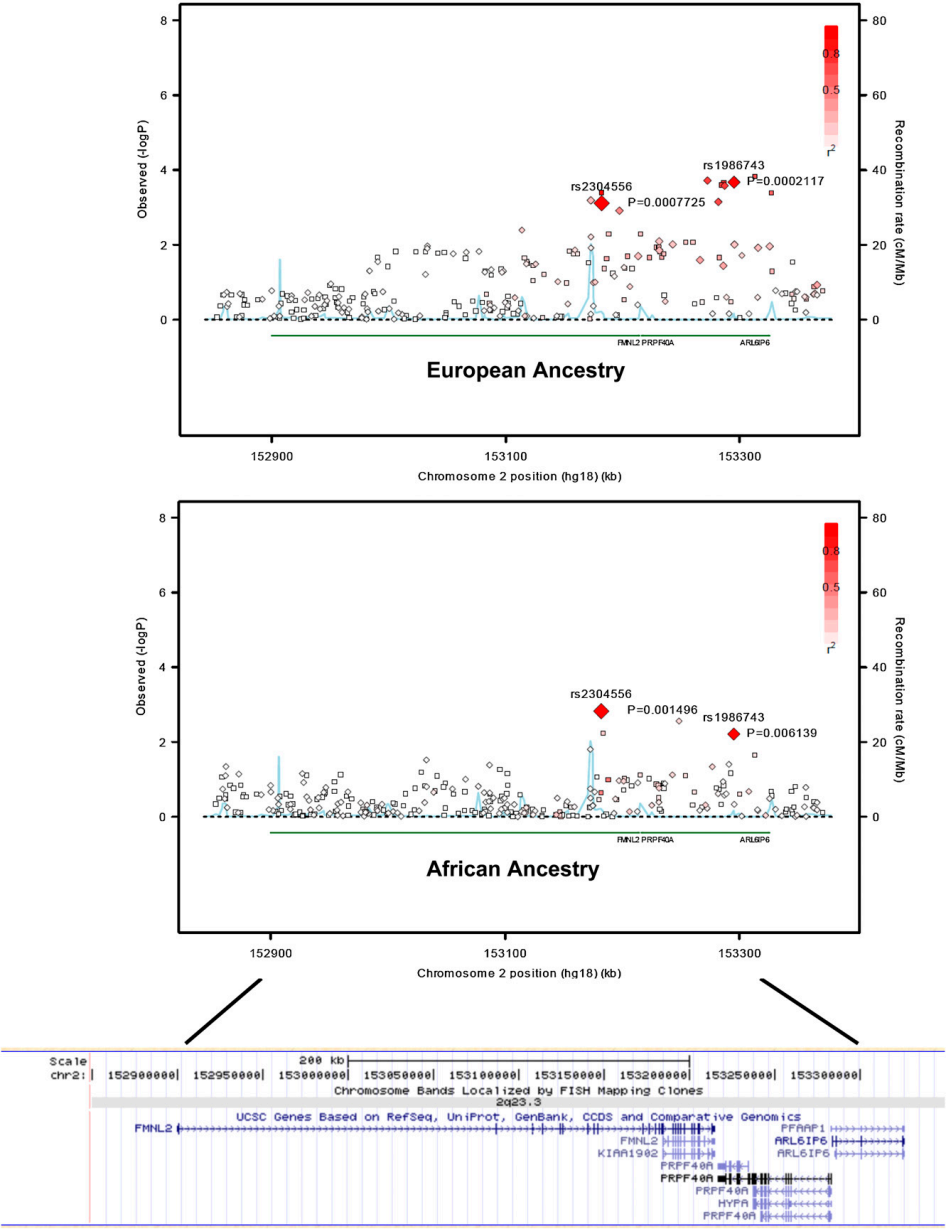
Regional association plots showing associations of FMNL2-ARL6IP6I region in European ancestry (top) and in African ancestry populations (middle), and the position of the genes in this region (bottom). Each diamond represents a single typed SNP, and each square represents a single imputed SNP. The color within each square/diamond represents the extent of LD correlation, r2, between the SNP and rs2304556. The blue line represents the recombination rate. Recombination rate and r^2^ were estimated based on HapMap3 CEU and YRI samples, respectively. Regional association plots were drawn by modifying the R program codes from the SNAP program, and the position of the gene were obtained from the University of California, Santa Cruz (UCSC) genome browser.

Results from the GWA analysis are summarized in Figure 1. The genomic inflation factor was 1.01, indicating that the distribution of observed *P* values across most of the genome was consistent with that expected under the null hypothesis. The 10 most strongly associated SNPs are listed in Table 3. No single SNP reached genome-wide significance (α = 5.0 × 10^−8^). However, we found SNPs, rs2304556 (*P* = 1.2 × 10^−7^) and rs1986743 (*P* = 2.7 × 10^−7^) located on chromosome 2q23.3, that were associated with IS but not at genome-wide significance (*P* < 5 × 10^−8^). These two SNPs are separated by 113 kb and are in strong linkage disequilibrium (LD) in EA (D′ = 0.90, r^2^ = 0.75) although not in AA participants (D′ = 0.35, r^2^ = 0.08) in our data [see Figure 2 for regional association plots for EA and AA obtained with the SNAP program (Johnson *et al.* 2008)]. The estimated OR for both SNPs was 0.69 (95% CI = 0.60–0.79) per copy of the minor allele (Table 3). The minor alleles of the two SNPs were more common in controls than cases and more common in AA than EA populations. The effects of the minor allele on risk of IS are similar between EA and AA populations for rs2304556 (OR= 0.71, *P* = 0.00077 in EA and OR = 0.71, *P* = 0.0015 in AA, allele G) and for rs1986743 (OR= 0.68, *P* = 0.0002 in EA and OR = 0.73, *P* = 0.006 in AA, allele A). After adjusting for the effect of rs2304556, the effect of rs1986743 became nonsignificant in EA (OR = 0.72, *P* = 0.11) and borderline significant in AA (OR = 0.79, *P* = 0.06). Additionally, we reexamined the associations of the two SNPs by further adjustment for prior history of MI, hypertension, diabetes and for current smoking, and the effects of the two SNPs remained similar (rs2304556: OR = 0.68, 95% CI = 0.59∼0.79; *P* = 1.9 × 10^−7^; rs1986743: OR = 0.70, 95% CI = 0.61∼0.82; *P* = 3.6 × 10^−6^). rs2304556 is in the intron of *FMNL2*, which encodes a formin-related protein, and rs1986743 is in the intron of *ARL6IP6*, which encodes ADP-ribosylation-like factor 6 interacting protein 6. The cluster plots of both SNPs (rs2304556 and rs1986743) also showed clear separation of the three genotypes, indicating good clustering of genotype calls for the two SNPs (see Figure S2). Most of the other top-associated SNPs were located in intergenic regions, except rs9465922 in an intron of *CDKAL1* and rs16834810 in an intron of *RYR2*.

**Table 3:** Association results of the top 10 SNPs based on results of overall population, ranked by *P* values

					Effect Allele Frequency								
					European Ancestry*^a^*			African Ancestry*^a^*			Combined		
SNP	Chr	Position*^b^*	Imp*^c^*	Effect/Noneffect Allele*^d^*	Case	Control	*P*	Case	Control	*P*	OR (95% CI)*^e^*	*P*	Closest Gene*^f^*
rs2304556	2	153182040	No	G/T	0.28	0.36	7.7E-04	0.40	0.48	1.5E-03	0.69 (0.60, 0.79)	1.2E-07	*FMNL2* (intron)
rs1986743	2	153295145	No	A/G	0.30	0.38	2.1E-04	0.31	0.37	6.1E-03	0.69 (0.60, 0.79)	2.7E-07	*ARL6IP6* (intron)
rs3909263	13	71753222	Yes	C/G	0.26	0.31	1.4E-02	0.35	0.46	2.9E-06	0.68 (0.59, 0.79)	7.3E-07	*DACH1* (413.9 kb)
rs1146849	13	71770207	No	A/G	0.24	0.29	2.2E-02	0.36	0.47	3.3E-06	0.7 (0.61, 0.81)	1.7E-06	*DACH1* (430.9 kb)
rs17366217	5	61530870	No	T/C	0.08	0.11	5.5E-03	0.15	0.22	5.0E-04	0.61 (0.5, 0.75)	2.0E-06	*KIF2A* (147.8 kb)
rs879012	20	957788	Yes	C/T	0.38	0.29	1.7E-05	0.48	0.41	2.3E-02	1.43 (1.23, 1.65)	2.2E-06	*RSPO4* (26.9 kb)
rs9465922	6	20973576	No	C/A	0.03	0.07	4.9E-04	0.14	0.20	1.8E-03	0.57 (0.45, 0.72)	2.3E-06	*CDKAL1* (intron)
rs2605877	8	74309320	No	A/G	0.37	0.27	5.8E-06	0.37	0.32	4.0E-02	1.41 (1.22, 1.64)	3.0E-06	*RPL7* (56.1 kb)
rs16834810	1	235325422	Yes	A/G	0.01	0.03	3.4E-03	0.02	0.06	2.6E-04	0.3 (0.18, 0.5)	3.3E-06	*RYR2* (intron)
rs9604365	13	111625198	No	G/A	0.06	0.05	5.0E-01	0.52	0.39	8.1E-07	1.54 (1.28, 1.86)	3.9E-06	*SOX1* (144.7 kb)

Abbreviations: Chr, chromosome; CI, confidence interval; Imp, Imputation; OR, odds ratio; *P*, association *P* value.

a European ancestry and African ancestry are defined based on multidimensional scaling (MDS) analysis.

b Physical position is based on National Center for Biotechnology Information (NCBI) build 36.

c Flag indicating whether the SNP was imputed (Yes) or directly genotyped (No). The total number of individuals in the combined analysis is 1816 for directly genotyped SNPs and 1679 for imputed SNPs.

d Polymorphism is reported based on genome assembly plus strand.

e Odds ratio per copy of effect allele.

f The closest gene to the SNP and the location/distance of the SNP to the gene.

No SNPs achieved genome-wide significance in ethnic group–specific analyses (data not shown). In the EA population, the SNP most strongly associated with IS was rs2242878 at *RUNX1* (OR = 1.81, 95% CI = 1.42–2.31, *P* = 1.8 × 10^−6^, T allele), but this SNP was not significantly associated with IS in the AA population (OR = 0.95, 95% CI = 0.55–1.65, *P* = 0.87). Similarly, the SNP most strongly associated with IS in AAs was rs9604365 near *SOX1* (OR = 1.74, 95% CI = 1.40–2.17, *P* = 8.1 × 10^−7^, allele G), but this SNP was not associated with IS in EA (OR= 1.15, 95% CI = 0.77–1.72, *P* = 0.5). It should be noted that rs9604365 was the tenth most significant SNP based on the overall population results (see Table 3).

### Genome-wide association of stroke subtypes

We further analyzed the genome-wide associations stratified by each stroke subtype defined by TOAST criteria, including cardioembolic, large artery, lacunar subtypes, other known causes, and other undetermined causes. No SNPs were associated with any of the stroke subtypes at genome-wide levels of statistical significance, although the power to detect associations for subtypes was very modest. Summary results showing all SNPs associated with any stroke subtype at *P* < 1.0 × 10^−5^ in combined analyses of EA and AA are provided in Table S1. Neither rs2304556 nor rs1986743, the two SNPs most strongly associated with overall stroke in our sample, showed subtype-specific patterns of association; *i.e.*, the associations for both SNPs were approximately consistent across all stroke subtypes (ORs ranged 0.52–0.76 for rs2304556 and 0.57–0.73 for rs1986743) (Table S2).

### Replications of chromosome 2 SNP associations in other Caucasian cohorts

There are currently no other large cohorts of young-onset stroke for which to attempt replication. We did, however, assess the association of rs2304556, our peak SNP, with IS in two independent Caucasian cohorts with older-onset stroke: the Ischemic Stroke Genetics Study and Siblings with Ischemic Stroke Studies (ISGS/SWISS; n = 1070 cases and 1488 controls) and the Genes Affecting Stroke Risk and Outcomes Study (GASROS; n = 516 cases and 1202 controls). There was no evidence for association with IS in either cohort (OR = 1.10, *P* = 0.12 in ISGS/SWISS and OR = 0.95, *P* = 0.63 in GASROS for minor allele G). These cohorts had 80% power to detect odds ratios as low as 1.21 (ISGS/SWISS) and 1.26 (GASROS) for this SNP.

### Associations of *NINJ2* variants candidate genes with early-onset IS

We additionally tested whether SNPs from *NINJ2*, which was identified in a prior large GWAS study to be associated with IS, were associated with young-onset stroke in our study (Table 4). Of the two SNPs near *NINJ2* that were previously reported to be associated with IS (Ikram *et al.* 2009), neither was associated with early-onset IS in our samples (rs11833579, combined *P* = 0.54, EA *P* = 0.99, AA *P* = 0.76; and rs12425791, combined *P* = 0.49, EA *P* = 0.74, AA *P* = 0.91). None of the other SNPs from *NINJ2* was significantly associated with stroke risk in EA and AA populations after controlling for the number of SNPs analyzed within the gene. When further investigating the *NINJ2* associations by TOAST subtypes, none of the SNPs was significantly associated with any of the stroke subtype, either (data not shown).

**Table 4:** Association results of *NINJ2* in the GEOS Study

		Previously Reported Variant				Best SNP within the Gene				
	No. of SNPs*^a^*	Variant	Effect/Noneffect Allele	OR (95% CI)	*P**^b^*	Variant	Effect/Noneffect Allele	OR (95% CI)	*P**^b^*	Empirical *P**^c^*
EA*^d^*	70	rs12425791	A/G	0.96 (0.76, 1.21)	0.74	rs12229103	A/C	1.34 (0.90, 2.00)	0.15	0.95
		rs11833579	A/G	1.00 (0.81, 1.24)	0.99					
AA*^d^*	70	rs12425791	A/G	1.02 (0.73, 1.42)	0.91	rs11063749	T/C	0.74 (0.60, 0.93)	0.008	0.27
		rs11833579	A/G	1.04 (0.80, 1.35)	0.76					

Abbreviations: CI, confidence interval; OR, odds ratio; *P*, association *P* value.

a Number of SNPs within the gene that were either directly genotyped (SNP call rate > 95% and HWE *P* > 1.0 × 10^−7^ in either European or African ancestry) or imputed (imputation dosage r2 > 0.3).

b Nominal *P* was obtained by t-statistics in the logistic regression.

c Empirical *P* was obtained by permutations as described in *Materials and Methods*.

d European ancestry (EA) and African ancestry (AA) are defined based on multidimensional scaling (MDS) analysis.

## Discussion

### Suggestive associations between chromosome 2q23.3 and early-onset IS

The GEOS Study is the largest genetics study of young-onset stroke carried out to date. Despite having 80% power to detect odds ratios of 1.55–2.30 (at allele frequencies 10–50%), we had limited power (<70%) to detect odds ratio less than 1.5 and failed to identify associations with SNPs meeting conventional genome-wide levels of significance. However, we did detect associations with two SNPs on chromosome 2q associated with overall IS that achieved nearly genome-wide levels of significance (*P* < 2.65 × 10^−7^). The most strongly associated SNPs were near *FMNL2* on chromosome 2. The minor allele of this SNP (rs2304556), which is located in the intron of *FMNL2*, is associated with an approximately 31% decrease in risk per copy of minor allele in both EA and AA populations. A similar association with stroke risk was observed for a nearby SNP, rs1986743, located on *ARL6IP6*, although the strength of association for this SNP diminished substantially after adjusting for rs2304556, suggesting the two SNPs may not represent independent signals. As there are no other large studies of young strokes currently available for replication, we sought replication in two other cohorts with older strokes (ISGS and GASROS). Although we were unable to replicate the associations of the chromosome 2 SNPs in other Caucasian cohorts, the lack of replications may be due to the genetic heterogeneity possibility between younger *vs.* older strokes.

*FMNL2*, which encodes formin-like 2 protein, is involved in actin-dependent processes and is implicated in cell motility and invasion (Kitzing *et al.* 2010). Although the function of this gene is largely unknown, the gene is highly expressed in the brain and other central/peripheral nervous system tissues (http://www.cgl.ucsf.edu/Research/genentech/genehub-gepis/index.html) (Gardberg *et al.* 2010) and is conserved in different species (http://www.ncbi.nlm.nih.gov/sites/homologene). A microdeletion on chromosome 2q region involving *FMNL2* has previously been implicated in mental retardation (Lybæk *et al.* 2009), and association and linkage studies have implicated this same chromosomal region with susceptibility to autism (Imgsac. 2001; Maestrini *et al.* 2009). The function of *ARL6IP6* is currently unknown. Replication studies, particularly in young stroke cases, are required to assess the role of rs2304556 and/or other variants in and around *FMNL2* with young-onset stroke.

Interestingly, our GWAS also showed *CDKAL1*, a gene previously shown to be associated with type 2 diabetes (T2D) (Scott *et al.* 2007; Zeggini *et al.* 2007), to be marginally associated with IS in our data. T2D is a strong risk factor for stroke. However, the association between rs9465922 and IS remained highly significant after adjusting for self-reported diabetes status in our population (OR = 0.55, 95% = 0.44–0.70, *P* = 5.5 × 10^−7^), suggesting the effect of this gene on IS may be independent of the effect of T2D. The function of *CDKAL1* is not currently known, although the association of this gene to T2D may be related to an effect of the associated SNP on insulin response (Steinthorsdottir *et al.* 2007).

### Failure to replicate previously reported associations with *NINJ2*

We were unable to replicate the previous GWA associations with two SNPs in *NINJ2* as reported by the CHARGE consortium (Ikram *et al.* 2009), even though power was 98% in our sample to detect associations of the magnitude of that observed in CHARGE (OR = 1.39–1.41 for SNPs with these allele frequencies). Notably, the association with *NINJ2* reported in CHARGE could also not be replicated in a recent very large meta-analysis by the International Stroke Genetics Consortium and Wellcome Trust Case-Control Consortium 2 (2010).

### Strengths and limitations of the study

We used the strategy of studying the early-onset form of IS to identify genetic variants associated with this complex disease because the early-onset form may be less susceptible to the influence of environmental exposures, and genetic components could potentially have a greater contribution to disease risk. Although it is possible that genetic variants associated with early-onset stroke may have little relevance to late-onset stroke, it is also possible that studying early-onset form of diseases may reveal important insights about stroke pathogenesis, even implicating key genes and pathways relevant to late-onset disease even though the responsible variants may differ.

Although we did identify a region on chromosome 2 with suggestive evidence for association in the GEOS population, we did not identify any variants reaching conventional thresholds for statistical significance at a genome-wide level. The absence of robust associations may be due to the limited sample size that provided insufficient power to detect causal variants with small effect sizes (*e.g.* less than 80% power to detect genetic risk effect less than 1.55). Alternatively, one can argue that the early-onset form of IS may be more likely to be caused by rare variants with larger effects (and higher penetrance). Such rare variants are generally not well covered on currently available genotyping platforms and, thus, would be difficult to detect through a GWA approach. Exome and/or whole-genome sequencing may be a promising research approach to pursue the roles of these infrequent and highly penetrant variants on IS risk. Notably, our ability to identify IS-associated variants could be further limited by the stroke phenotype itself, which is composed of several heterogeneous subtypes with different underlying causes and potentially different genetic dispositions (Marnane *et al.* 2010). Thus, the genetic variants contributing to stroke risk may differ based upon stroke subtype. Although we analyzed according to TOAST subtype, in doing so we incurred further losses in power due to the smaller number of cases available in each subtype. As IS occurs infrequently before age 50, it is difficult for a single study to obtain the sufficient number of strokes necessary to power subtype-specific analyses. Therefore, combining genetic data among multiple early-onset stroke studies to increase the number of cases for overall stroke and stroke subtype analyses will be essential to ensure sufficient power to detect causal variants with moderate effects.

### Conclusions

Our early-onset IS GWA study identified a novel IS susceptibility region around *FMNL2* on chromosome 2q. Further studies in other young-stroke populations are needed to replicate these findings and to evaluate the associations between these SNPs and the more common older-onset form of stroke. Meta-analysis of early-onset stroke ensuring sufficient statistical power for moderate genetic effects and sequencing efforts to explore the effect of rare variants are also required.

## Supplementary Material

Supporting Information
