# Supplementary material for: Genome-Wide Association Analysis of Ischemic Stroke in Young Adults
Source: G3 (Bethesda). 2011 Nov 1;1(6):505–14. doi: 10.1534/g3.111.001164 (PMC3276159; doi:10.1534/g3.111.001164)
Supplement: Supporting Information [file supp_1_6_505__index.html]

Supporting Information 

# Genome-Wide Association Analysis of Ischemic Stroke in Young Adults

## Supporting Infomation for Cheng *et al.*, 2011

**Files in this Data Supplement:**

- Supporting Information - Figures S1 and S2 and Table S1 and S2 (PDF, 444 KB)
- Figure S1 - Imputation quality as measured by analysis of a masked set of SNPs on chromosome 1 for EA (panel A) and AA (panel B) study subjects (PDF, 108 KB)
- Figure S2 - Cluster plots of rs2304556 and rs1986743 showing the polar coordinate angle of a sample-SNP combination point (theta) and the sum of the two allelic intensities (R) calculated based on the entire samples (PDF, 156 KB)
- Table S1 - SNPs associated with each TOAST subtype with P < 0.00001 in the combined samples of GEOS, sorted by p-values (PDF, 148 KB)
- Table S2 - The association results of the two most strongly associated SNPs on chromosome 2, stratified by TOAST subtype (PDF, 48 KB)
