# Supplementary material for: Genome-Wide Association Analysis of Ischemic Stroke in Young Adults
Source: G3 (Bethesda). 2011 Nov 1;1(6):505–14. doi: 10.1534/g3.111.001164 (PMC3276159; doi:10.1534/g3.111.001164)
Supplement: Supporting Information [file supp_1.6.505_001164SI.pdf]

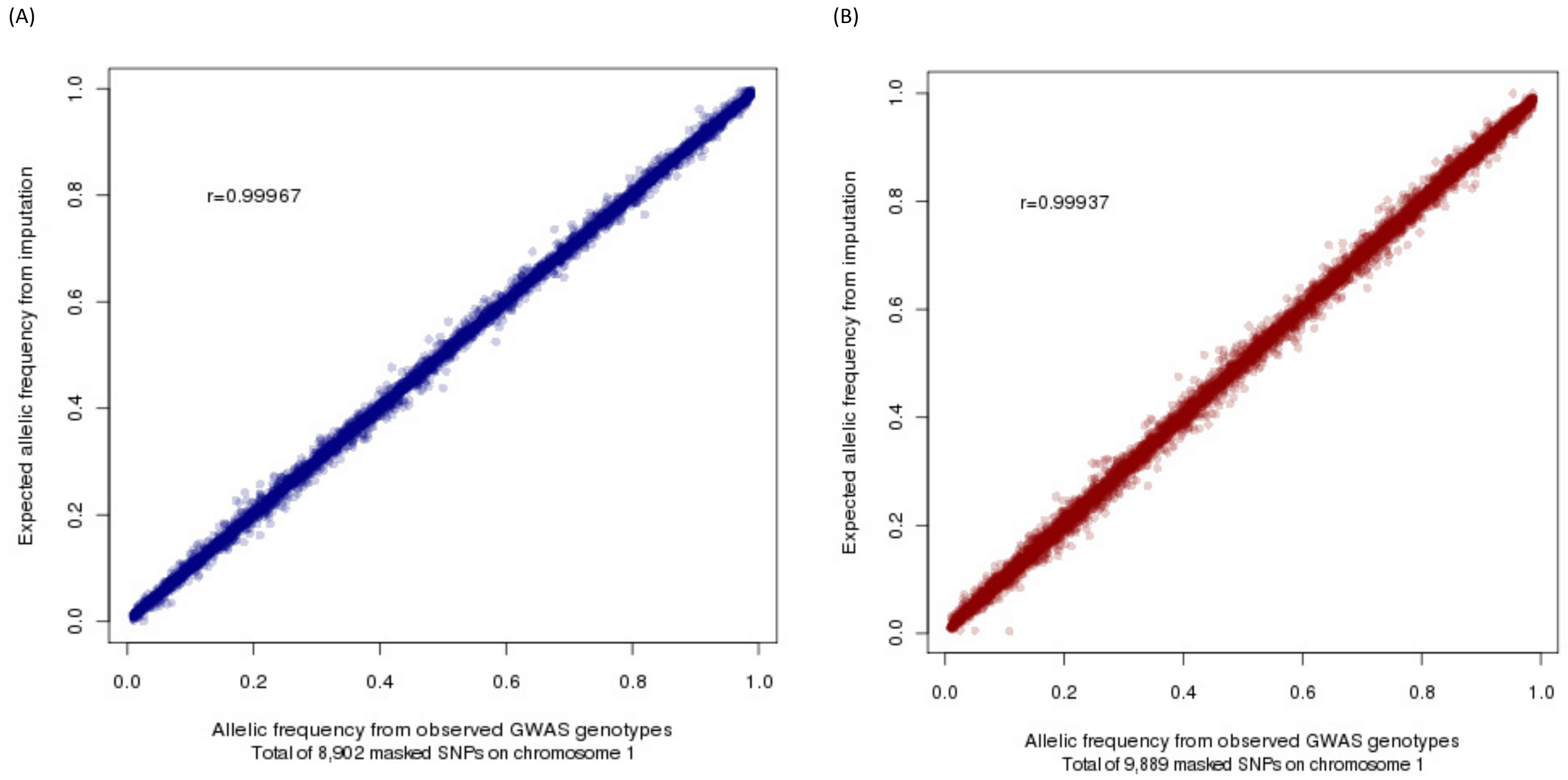

Figure S1 Imputation quality as measured by analysis of a masked set of SNPs on chromosome 1 for EA (panel A) and AA (panel B) study subjects. Each data point represents the expected and observed frequency of the reference allele for each masked SNP. The x-axis represents the allelic frequency estimated from the experimentally observed genotypes. The y-axis represents the expected allelic frequency based on each individual's expected allelic dosage as estimated from the masked imputation analysis.

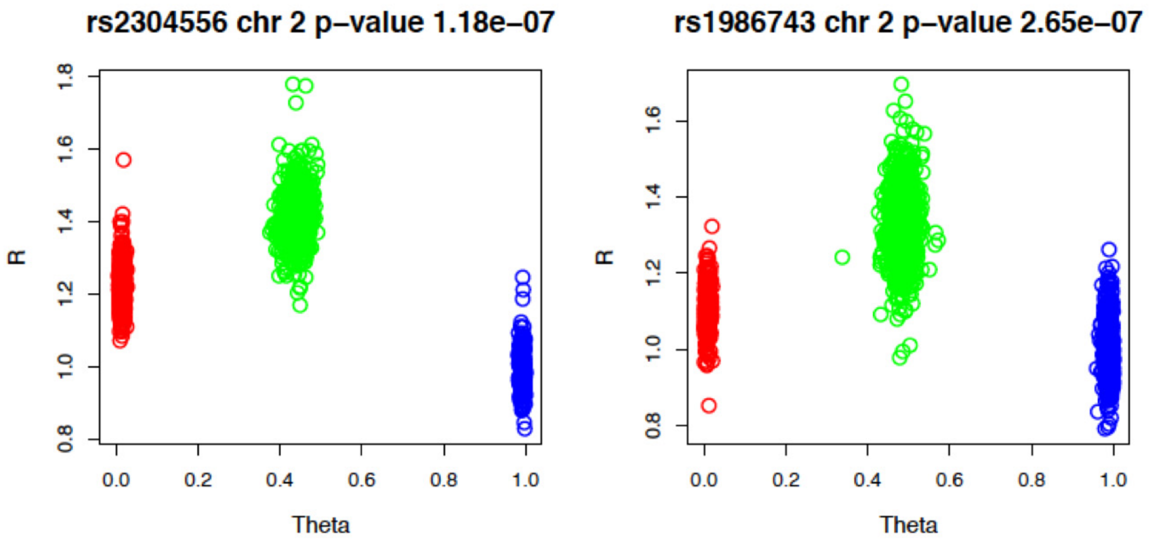

Figure S2 Cluster plots of rs2304556 and rs1986743 showing the polar coordinate angle of a sample-SNP combination point (theta) and the sum of the two allelic intensities (R) calculated based on the entire samples.

Table S1 SNPs associated with each TOAST subtype with P < 0.00001 in the combined samples of GEOS, sorted by p-values

SNP	Chr	Position	Effect/ Non-effect allele[b]	European Ancestry[a]			African Ancestry[a]			Overall	Nearby Gene(s)
				EAF[c]	OR (95% CI)	*P*	EAF[c]	OR (95% CI)	*P*	*P*	
**Cardioembolic**					90 cases/498 controls			74 cases/ 352 controls			
rs317838	18	53489654	A/G	0.34	1.77 (1.26, 2.49)	9.6E-04	0.35	2.06 (1.39, 3.05)	3.2E-04	2.2E-06	*ATP8B1*
rs317835	18	53490809	A/C	0.34	1.78 (1.26, 2.5)	9.4E-04	0.35	2.06 (1.39, 3.04)	3.3E-04	2.2E-06	*ATP8B1*
rs11107070	12	92446148	A/G	0.71	2.29 (1.47, 3.57)	2.4E-04	0.81	2.38 (1.31, 4.35)	4.7E-03	3.9E-06	*MRPL42/ SOCS2*
rs10191803	2	192743968	C/T	0.69	0.68 (0.48, 0.95)	2.5E-02	0.68	0.41 (0.27, 0.62)	1.9E-05	5.8E-06	*TMEFF2*
rs220282	21	42366825	A/G	0.22	2.02 (1.43, 2.84)	5.6E-05	0.33	1.47 (1.01, 2.13)	4.6E-02	8.1E-06	*UMODL1*
rs10732643	12	87686246	T/G	0.05	1.43 (0.72, 2.85)	3.1E-01	0.67	2.89 (1.78, 4.71)	2.0E-05	8.3E-06	*KITLG*
**Large Artery**					37 cases/498 controls			23 cases/ 352 controls			
rs6095892	20	48419278	C/T	0.1	3.21 (1.63, 6.33)	7.7E-04	0.42	3.15 (1.59, 6.22)	9.9E-04	6.4E-07	*COX6CP2*
rs2283436	15	87532780	C/T	0.07	6.95 (3.22, 15)	7.7E-07	0.14	2.43 (1.11, 5.31)	2.6E-02	7.0E-07	*ABHD2*
rs7266895	20	48415922	A/G	0.1	3.27 (1.66, 6.46)	6.3E-04	0.41	3.01 (1.54, 5.87)	1.3E-03	1.2E-06	*COX6CP2*
rs7506112	18	70413022	C/T	0.06	6.53 (2.9, 14.72)	6.0E-06	0.01	4.74 (0.86, 26.19)	7.4E-02	1.7E-06	*CNDP1*
rs7700699	5	85655321	A/T	0.97	0.22 (0.07, 0.69)	9.6E-03	0.8	0.25 (0.13, 0.49)	7.0E-05	2.9E-06	*NBPF22P*

rs1994487	3	87345688	G/A	0.03	4.38 (1.73, 11.06)	1.8E-03	0.22	2.58 (1.37, 4.89)	3.5E-03	4.8E-06	*CHMP2B*
rs35405829	13	95399680	T/C	0.02	4.59 (1.5, 14.07)	7.7E-03	0.08	3.49 (1.52, 8.01)	3.2E-03	5.7E-06	*UGCGL2*
rs7164909	15	56474046	T/C	0.09	3.54 (1.76, 7.11)	3.8E-04	0.1	2.83 (1.29, 6.21)	9.3E-03	5.9E-06	*LIPC*
rs4238329	15	56475478	C/A	0.07	3.91 (1.91, 7.99)	1.8E-04	0.09	2.51 (1.13, 5.54)	2.3E-02	7.6E-06	*LIPC*
rs10069795	5	85577682	C/T	0.03	5.14 (1.6, 16.5)	6.0E-03	0.21	3.51 (1.82, 6.75)	1.8E-04	8.0E-06	*NBPF22P*
rs986027	1	48485981	C/T	0.2	0.14 (0.04, 0.45)	1.2E-03	0.53	0.41 (0.21, 0.81)	9.5E-03	9.1E-06	*SLC5A9*
**Lacunar**					54 cases/498 controls			77 cases/352 controls			
rs11579489	1	15663226	A/G	0.07	2.16 (1.12, 4.16)	2.2E-02	0.1	2.73 (1.57, 4.75)	3.6E-04	3.0E-06	*ELA2A*
rs17067774	13	76936809	A/C	0.03	1.36 (0.41, 4.5)	6.1E-01	0.16	3.02 (1.92, 4.77)	1.9E-06	3.8E-06	*SCEL/ MYCBP2*
rs12029840	1	114333003	T/C	0.1	2.83 (1.59, 5.05)	4.3E-04	0.08	2.65 (1.43, 4.93)	2.0E-03	4.3E-06	*OLFML3*
rs12429367	13	76934513	C/T	0.97	0.78 (0.23, 2.59)	6.8E-01	0.84	0.33 (0.21, 0.52)	1.9E-06	4.3E-06	*SCEL/ MYCBP2*
rs910313	14	28268236	G/A	0.23	0.47 (0.25, 0.87)	1.7E-02	0.48	0.48 (0.32, 0.73)	6.8E-04	6.5E-06	*BTF3P2/ FOXG1*
rs11062697	12	3501629	A/C	0.09	2.61 (1.52, 4.49)	5.4E-04	0.15	1.8 (1.07, 3.04)	2.7E-02	8.5E-06	*PRMT8*
rs2388712	10	8422509	A/G	0.14	3.32 (1.84, 6)	6.7E-05	0.05	2.76 (0.77, 9.82)	1.2E-01	9.1E-06	*KRT8P16/*

											GATA3
**Other Known Causes**					29 cases/498 controls			74 cases/352 controls			
rs12417971	11	68726384	T/C	0.03	5.88 (2.22, 15.59)	3.7E-04	0.09	5.13 (2.29, 11.49)	7.1E-05	5.9E-07	*TPCN2/ MYEOV*
rs10897466	11	63479814	G/A	0.42	2.73 (1.43, 5.18)	2.2E-03	0.25	2.47 (1.32, 4.61)	4.7E-03	1.4E-06	*NAT11*
rs12798491	11	63471506	C/A	0.42	2.75 (1.45, 5.21)	1.9E-03	0.25	2.44 (1.3, 4.58)	5.3E-03	1.9E-06	*NAT11*
rs4238241	13	104717057	A/C	0.88	0.33 (0.16, 0.71)	4.3E-03	0.9	0.28 (0.13, 0.59)	7.9E-04	2.3E-06	*DAOA*
rs6910142	6	150614023	C/T	0.05	5.52 (2.29, 13.31)	1.4E-04	0.04	3.88 (1.35, 11.15)	1.2E-02	2.4E-06	*PPP1R14C*
rs11789156	9	110560665	G/A	0.13	3.21 (1.54, 6.68)	1.8E-03	0.07	3.28 (1.27, 8.48)	1.4E-02	9.4E-06	*ACTL7B*
**Other Undetermined Causes**					238 cases/498 controls			183 cases/352 controls			
rs7549251	1	55077558	G/A	0.35	1.36 (1.08, 1.71)	9.6E-03	0.56	1.86 (1.42, 2.44)	7.8E-06	1.8E-06	C1orf177
rs13259157	8	17527308	A/G	0.21	0.49 (0.35, 0.67)	1.2E-05	0.59	0.7 (0.53, 0.92)	1.1E-02	2.1E-06	PDGFRL
rs11730667	4	74819075	A/G	0.59	0.67 (0.54, 0.84)	4.0E-04	0.28	0.58 (0.42, 0.82)	1.8E-03	2.6E-06	IL8
rs67383011	4	184849547	C/G	0.12	0.37 (0.23, 0.59)	2.8E-05	0.05	0.49 (0.24, 1)	5.1E-02	3.0E-06	C4orf41
rs2239354	16	55976488	A/G	0.08	1.6 (1.11, 2.31)	1.2E-02	0.09	2.13 (1.46, 3.11)	9.3E-05	3.8E-06	CX3CL1
rs390783	3	8208002	A/G	0.09	1.77 (1.27, 2.46)	7.9E-04	0.28	1.51 (1.15, 1.99)	3.3E-03	3.9E-06	GRM7/ LMCD1

rs2074132	7	111323099	C/T	0.42	0.61 (0.48, 0.78)	6.0E-05	0.81	0.7 (0.51, 0.97)	3.4E-02	5.4E-06	DOCK4
rs10228638	7	111318884	C/T	0.42	0.61 (0.48, 0.78)	6.8E-05	0.81	0.7 (0.5, 0.97)	3.0E-02	5.4E-06	DOCK4
rs757036	7	111305649	A/G	0.41	0.63 (0.49, 0.8)	1.4E-04	0.72	0.69 (0.53, 0.91)	7.9E-03	5.8E-06	DOCK4
rs13088606	3	8205904	T/C	0.08	1.67 (1.16, 2.41)	5.9E-03	0.16	1.73 (1.26, 2.38)	7.0E-04	6.1E-06	GRM7/ LMCD1
rs2708252	7	147285314	G/A	0.09	1.67 (1.2, 2.32)	2.4E-03	0.25	1.49 (1.13, 1.95)	4.5E-03	6.7E-06	CNTNAP2
rs4694636	4	74817673	G/T	0.4	1.52 (1.21, 1.9)	2.5E-04	0.78	1.58 (1.12, 2.23)	9.1E-03	7.7E-06	IL8
rs6780741	3	8215179	G/A	0.09	1.77 (1.27, 2.46)	7.9E-04	0.31	1.44 (1.1, 1.88)	8.6E-03	9.1E-06	GRM7/ LMCD1
rs879012	20	957788	C/T	0.29	1.62 (1.28, 2.06)	7.4E-05	0.41	1.35 (1.03, 1.76)	2.7E-02	9.3E-06	RSPO4/ PSMF1
rs4684589	3	8214386	A/G	0.09	1.76 (1.26, 2.45)	8.5E-04	0.28	1.5 (1.14, 1.98)	3.9E-03	9.8E-06	GRM7/ LMCD1

Abbreviations: Chr: chromosome; EAF: effect allele frequency; OR: odds ratio; CI: confidence interval; *P:* association p-value

[a] European Ancestry and African Ancestry defined by MDS analysis

[b] Polymorphism is reported based on genome assembly PLUS strand

[c] Allele frequency is calculated based on controls

Table S2 The association results of the two most strongly associated SNPs on chromosome 2, stratified by TOAST subtype

	Allele Frequency							
	European Ancestry [a]			African Ancestry [a]			Combined	
SNP	Case	Control	*P*	Case	Control	*P*	OR (95% CI)	*P*
**rs2304556 (G allele)**								
Cardioembolic	0.278	0.356	3.3E-02	0.460	0.483	5.8E-01	0.76 (0.60, 0.97)	2.6E-02
Large Artery	0.230	0.356	2.8E-02	0.348	0.483	1.1E-01	0.52 (0.34, 0.78)	1.7E-03
Lacunar	0.296	0.356	1.8E-01	0.396	0.483	6.0E-02	0.67 (0.51, 0.89)	4.9E-03
Other Know Causes	0.224	0.356	3.9E-02	0.313	0.483	3.7E-02	0.48 (0.30, 0.76)	2.0E-03
Undetermined Causes	0.298	0.356	2.2E-02	0.393	0.483	8.8E-03	0.72 (0.61, 0.85)	1.1E-04
**rs1986743 (A allele)**								
Cardioembolic	0.306	0.376	5.2E-02	0.311	0.372	1.5E-01	0.70 (0.54, 0.90)	5.0E-03
Large Artery	0.216	0.376	3.9E-03	0.326	0.372	7.4E-01	0.57 (0.38, 0.87)	9.2E-03
Lacunar	0.324	0.376	1.9E-01	0.312	0.372	3.1E-01	0.73 (0.55, 0.97)	3.3E-02
Other Know Causes	0.259	0.376	6.5E-02	0.208	0.372	1.4E-02	0.45 (0.28, 0.72)	1.0E-03
Undetermined Causes	0.307	0.376	5.8E-03	0.309	0.372	4.3E-02	0.73 (0.61, 0.87)	3.9E-04

Abbreviations: OR: odds ratio; CI: confidence interval; *P:* association p-value
[a] European Ancestry and African Ancestry defined by MDS analysis
