# Supplementary material for: Genome-Wide Association Analysis of Ischemic Stroke in Young Adults
Source: G3 (Bethesda). 2011 Nov 1;1(6):505–14. doi: 10.1534/g3.111.001164 (PMC3276159; doi:10.1534/g3.111.001164)
Supplement: Supporting Information [file supp_1.6.505_FigureS1.pdf]

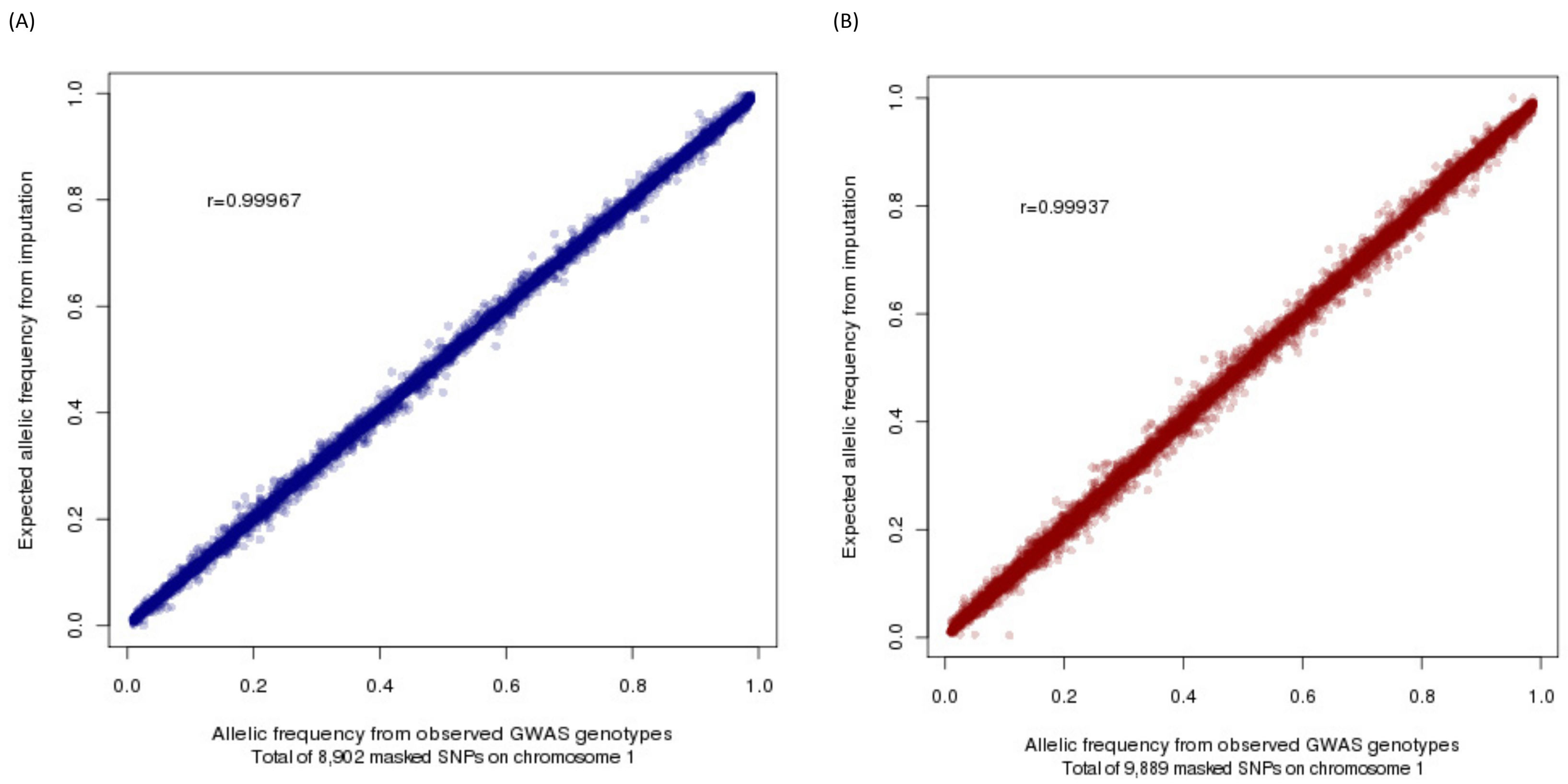

Figure S1 Imputation quality as measured by analysis of a masked set of SNPs on chromosome 1 for EA (panel A) and AA (panel B) study subjects. Each data point represents the expected and observed frequency of the reference allele for each masked SNP. The x-axis represents the allelic frequency estimated from the experimentally observed genotypes. The y-axis represents the expected allelic frequency based on each individual's expected allelic dosage as estimated from the masked imputation analysis.
