# Supplementary material for: Genome-Wide Association Analysis of Ischemic Stroke in Young Adults
Source: G3 (Bethesda). 2011 Nov 1;1(6):505–14. doi: 10.1534/g3.111.001164 (PMC3276159; doi:10.1534/g3.111.001164)
Supplement: Supporting Information [file supp_1.6.505_FigureS2.pdf]

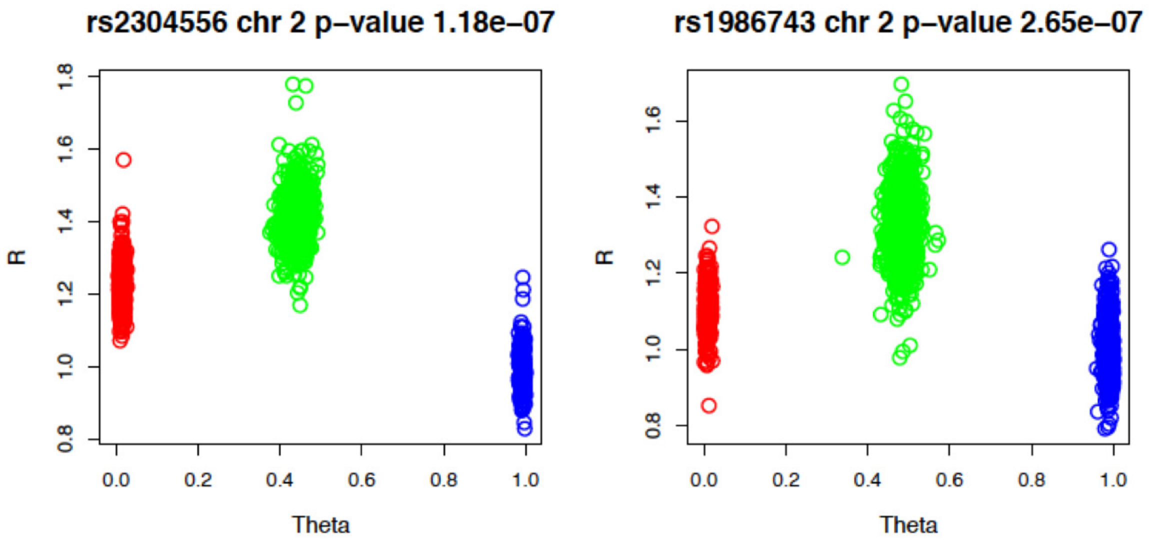

Figure S2 Cluster plots of rs2304556 and rs1986743 showing the polar coordinate angle of a sample-SNP combination point (theta) and the sum of the two allelic intensities (R) calculated based on the entire samples.
