# Supplementary material for: Genome-Wide Association Analysis of Ischemic Stroke in Young Adults
Source: G3 (Bethesda). 2011 Nov 1;1(6):505–14. doi: 10.1534/g3.111.001164 (PMC3276159; doi:10.1534/g3.111.001164)
Supplement: Supporting Information [file supp_1.6.505_TableS1.pdf]

Table S1 SNPs associated with each TOAST subtype with P < 0.00001 in the combined samples of GEOS, sorted by p-values

SNP	Chr	Position	Effect/ Non-effect allele[b]	European Ancestry[a]			African Ancestry[a]			Overall	Nearby Gene(s)
				EAF[c]	OR (95% CI)	*P*	EAF[c]	OR (95% CI)	*P*	*P*	
**Cardioembolic**					90 cases/498 controls			74 cases/ 352 controls			
rs317838	18	53489654	A/G	0.34	1.77 (1.26, 2.49)	9.6E-04	0.35	2.06 (1.39, 3.05)	3.2E-04	2.2E-06	*ATP8B1*
rs317835	18	53490809	A/C	0.34	1.78 (1.26, 2.5)	9.4E-04	0.35	2.06 (1.39, 3.04)	3.3E-04	2.2E-06	*ATP8B1*
rs11107070	12	92446148	A/G	0.71	2.29 (1.47, 3.57)	2.4E-04	0.81	2.38 (1.31, 4.35)	4.7E-03	3.9E-06	*MRPL42/ SOCS2*
rs10191803	2	192743968	C/T	0.69	0.68 (0.48, 0.95)	2.5E-02	0.68	0.41 (0.27, 0.62)	1.9E-05	5.8E-06	*TMEFF2*
rs220282	21	42366825	A/G	0.22	2.02 (1.43, 2.84)	5.6E-05	0.33	1.47 (1.01, 2.13)	4.6E-02	8.1E-06	*UMODL1*
rs10732643	12	87686246	T/G	0.05	1.43 (0.72, 2.85)	3.1E-01	0.67	2.89 (1.78, 4.71)	2.0E-05	8.3E-06	*KITLG*
**Large Artery**					37 cases/498 controls			23 cases/ 352 controls			
rs6095892	20	48419278	C/T	0.1	3.21 (1.63, 6.33)	7.7E-04	0.42	3.15 (1.59, 6.22)	9.9E-04	6.4E-07	*COX6CP2*
rs2283436	15	87532780	C/T	0.07	6.95 (3.22, 15)	7.7E-07	0.14	2.43 (1.11, 5.31)	2.6E-02	7.0E-07	*ABHD2*
rs7266895	20	48415922	A/G	0.1	3.27 (1.66, 6.46)	6.3E-04	0.41	3.01 (1.54, 5.87)	1.3E-03	1.2E-06	*COX6CP2*
rs7506112	18	70413022	C/T	0.06	6.53 (2.9, 14.72)	6.0E-06	0.01	4.74 (0.86, 26.19)	7.4E-02	1.7E-06	*CNDP1*
rs7700699	5	85655321	A/T	0.97	0.22 (0.07, 0.69)	9.6E-03	0.8	0.25 (0.13, 0.49)	7.0E-05	2.9E-06	*NBPF22P*

rs1994487	3	87345688	G/A	0.03	4.38 (1.73, 11.06)	1.8E-03	0.22	2.58 (1.37, 4.89)	3.5E-03	4.8E-06	*CHMP2B*
rs35405829	13	95399680	T/C	0.02	4.59 (1.5, 14.07)	7.7E-03	0.08	3.49 (1.52, 8.01)	3.2E-03	5.7E-06	*UGCGL2*
rs7164909	15	56474046	T/C	0.09	3.54 (1.76, 7.11)	3.8E-04	0.1	2.83 (1.29, 6.21)	9.3E-03	5.9E-06	*LIPC*
rs4238329	15	56475478	C/A	0.07	3.91 (1.91, 7.99)	1.8E-04	0.09	2.51 (1.13, 5.54)	2.3E-02	7.6E-06	*LIPC*
rs10069795	5	85577682	C/T	0.03	5.14 (1.6, 16.5)	6.0E-03	0.21	3.51 (1.82, 6.75)	1.8E-04	8.0E-06	*NBPF22P*
rs986027	1	48485981	C/T	0.2	0.14 (0.04, 0.45)	1.2E-03	0.53	0.41 (0.21, 0.81)	9.5E-03	9.1E-06	*SLC5A9*
**Lacunar**					54 cases/498 controls			77 cases/352 controls			
rs11579489	1	15663226	A/G	0.07	2.16 (1.12, 4.16)	2.2E-02	0.1	2.73 (1.57, 4.75)	3.6E-04	3.0E-06	*ELA2A*
rs17067774	13	76936809	A/C	0.03	1.36 (0.41, 4.5)	6.1E-01	0.16	3.02 (1.92, 4.77)	1.9E-06	3.8E-06	*SCEL/ MYCBP2*
rs12029840	1	114333003	T/C	0.1	2.83 (1.59, 5.05)	4.3E-04	0.08	2.65 (1.43, 4.93)	2.0E-03	4.3E-06	*OLFML3*
rs12429367	13	76934513	C/T	0.97	0.78 (0.23, 2.59)	6.8E-01	0.84	0.33 (0.21, 0.52)	1.9E-06	4.3E-06	*SCEL/ MYCBP2*
rs910313	14	28268236	G/A	0.23	0.47 (0.25, 0.87)	1.7E-02	0.48	0.48 (0.32, 0.73)	6.8E-04	6.5E-06	*BTF3P2/ FOXG1*
rs11062697	12	3501629	A/C	0.09	2.61 (1.52, 4.49)	5.4E-04	0.15	1.8 (1.07, 3.04)	2.7E-02	8.5E-06	*PRMT8*
rs2388712	10	8422509	A/G	0.14	3.32 (1.84, 6)	6.7E-05	0.05	2.76 (0.77, 9.82)	1.2E-01	9.1E-06	*KRT8P16/*

											GATA3
**Other Known Causes**					29 cases/498 controls			74 cases/352 controls			
rs12417971	11	68726384	T/C	0.03	5.88 (2.22, 15.59)	3.7E-04	0.09	5.13 (2.29, 11.49)	7.1E-05	5.9E-07	*TPCN2/ MYEOV*
rs10897466	11	63479814	G/A	0.42	2.73 (1.43, 5.18)	2.2E-03	0.25	2.47 (1.32, 4.61)	4.7E-03	1.4E-06	*NAT11*
rs12798491	11	63471506	C/A	0.42	2.75 (1.45, 5.21)	1.9E-03	0.25	2.44 (1.3, 4.58)	5.3E-03	1.9E-06	*NAT11*
rs4238241	13	104717057	A/C	0.88	0.33 (0.16, 0.71)	4.3E-03	0.9	0.28 (0.13, 0.59)	7.9E-04	2.3E-06	*DAOA*
rs6910142	6	150614023	C/T	0.05	5.52 (2.29, 13.31)	1.4E-04	0.04	3.88 (1.35, 11.15)	1.2E-02	2.4E-06	*PPP1R14C*
rs11789156	9	110560665	G/A	0.13	3.21 (1.54, 6.68)	1.8E-03	0.07	3.28 (1.27, 8.48)	1.4E-02	9.4E-06	*ACTL7B*
**Other Undetermined Causes**					238 cases/498 controls			183 cases/352 controls			
rs7549251	1	55077558	G/A	0.35	1.36 (1.08, 1.71)	9.6E-03	0.56	1.86 (1.42, 2.44)	7.8E-06	1.8E-06	C1orf177
rs13259157	8	17527308	A/G	0.21	0.49 (0.35, 0.67)	1.2E-05	0.59	0.7 (0.53, 0.92)	1.1E-02	2.1E-06	PDGFRL
rs11730667	4	74819075	A/G	0.59	0.67 (0.54, 0.84)	4.0E-04	0.28	0.58 (0.42, 0.82)	1.8E-03	2.6E-06	IL8
rs67383011	4	184849547	C/G	0.12	0.37 (0.23, 0.59)	2.8E-05	0.05	0.49 (0.24, 1)	5.1E-02	3.0E-06	C4orf41
rs2239354	16	55976488	A/G	0.08	1.6 (1.11, 2.31)	1.2E-02	0.09	2.13 (1.46, 3.11)	9.3E-05	3.8E-06	CX3CL1
rs390783	3	8208002	A/G	0.09	1.77 (1.27, 2.46)	7.9E-04	0.28	1.51 (1.15, 1.99)	3.3E-03	3.9E-06	GRM7/ LMCD1

rs2074132	7	111323099	C/T	0.42	0.61 (0.48, 0.78)	6.0E-05	0.81	0.7 (0.51, 0.97)	3.4E-02	5.4E-06	DOCK4
rs10228638	7	111318884	C/T	0.42	0.61 (0.48, 0.78)	6.8E-05	0.81	0.7 (0.5, 0.97)	3.0E-02	5.4E-06	DOCK4
rs757036	7	111305649	A/G	0.41	0.63 (0.49, 0.8)	1.4E-04	0.72	0.69 (0.53, 0.91)	7.9E-03	5.8E-06	DOCK4
rs13088606	3	8205904	T/C	0.08	1.67 (1.16, 2.41)	5.9E-03	0.16	1.73 (1.26, 2.38)	7.0E-04	6.1E-06	GRM7/ LMCD1
rs2708252	7	147285314	G/A	0.09	1.67 (1.2, 2.32)	2.4E-03	0.25	1.49 (1.13, 1.95)	4.5E-03	6.7E-06	CNTNAP2
rs4694636	4	74817673	G/T	0.4	1.52 (1.21, 1.9)	2.5E-04	0.78	1.58 (1.12, 2.23)	9.1E-03	7.7E-06	IL8
rs6780741	3	8215179	G/A	0.09	1.77 (1.27, 2.46)	7.9E-04	0.31	1.44 (1.1, 1.88)	8.6E-03	9.1E-06	GRM7/ LMCD1
rs879012	20	957788	C/T	0.29	1.62 (1.28, 2.06)	7.4E-05	0.41	1.35 (1.03, 1.76)	2.7E-02	9.3E-06	RSPO4/ PSMF1
rs4684589	3	8214386	A/G	0.09	1.76 (1.26, 2.45)	8.5E-04	0.28	1.5 (1.14, 1.98)	3.9E-03	9.8E-06	GRM7/ LMCD1

Abbreviations: Chr: chromosome; EAF: effect allele frequency; OR: odds ratio; CI: confidence interval; *P:* association p-value

[a] European Ancestry and African Ancestry defined by MDS analysis

[b] Polymorphism is reported based on genome assembly PLUS strand

[c] Allele frequency is calculated based on controls
