# Supplementary material for: Genome-Wide Association Analysis of Ischemic Stroke in Young Adults
Source: G3 (Bethesda). 2011 Nov 1;1(6):505–14. doi: 10.1534/g3.111.001164 (PMC3276159; doi:10.1534/g3.111.001164)
Supplement: Supporting Information [file supp_1.6.505_TableS2.pdf]

Table S2 The association results of the two most strongly associated SNPs on chromosome 2, stratified by TOAST subtype

	Allele Frequency							
	European Ancestry [a]			African Ancestry [a]			Combined	
SNP	Case	Control	*P*	Case	Control	*P*	OR (95% CI)	*P*
**rs2304556 (G allele)**								
Cardioembolic	0.278	0.356	3.3E-02	0.460	0.483	5.8E-01	0.76 (0.60, 0.97)	2.6E-02
Large Artery	0.230	0.356	2.8E-02	0.348	0.483	1.1E-01	0.52 (0.34, 0.78)	1.7E-03
Lacunar	0.296	0.356	1.8E-01	0.396	0.483	6.0E-02	0.67 (0.51, 0.89)	4.9E-03
Other Know Causes	0.224	0.356	3.9E-02	0.313	0.483	3.7E-02	0.48 (0.30, 0.76)	2.0E-03
Undetermined Causes	0.298	0.356	2.2E-02	0.393	0.483	8.8E-03	0.72 (0.61, 0.85)	1.1E-04
**rs1986743 (A allele)**								
Cardioembolic	0.306	0.376	5.2E-02	0.311	0.372	1.5E-01	0.70 (0.54, 0.90)	5.0E-03
Large Artery	0.216	0.376	3.9E-03	0.326	0.372	7.4E-01	0.57 (0.38, 0.87)	9.2E-03
Lacunar	0.324	0.376	1.9E-01	0.312	0.372	3.1E-01	0.73 (0.55, 0.97)	3.3E-02
Other Know Causes	0.259	0.376	6.5E-02	0.208	0.372	1.4E-02	0.45 (0.28, 0.72)	1.0E-03
Undetermined Causes	0.307	0.376	5.8E-03	0.309	0.372	4.3E-02	0.73 (0.61, 0.87)	3.9E-04

Abbreviations: OR: odds ratio; CI: confidence interval; *P:* association p-value
[a] European Ancestry and African Ancestry defined by MDS analysis
